# VLX1570 suppresses neuroblastoma growth through inhibition of PCNA-associated proliferative signaling and potentiates cisplatin antitumor activity

**DOI:** 10.3389/fphar.2026.1857567

**Published:** 2026-07-15

**Authors:** Wei-Hsun Yang, Chong-Sun Khoi, Kuan-Lin Kuo, Po-Ming Chow, Chen-Hsun Hsu, Yi-Ju Kao, Wei-Chou Lin, Shang-Jen Chang, Shih-Ming Liao, Felicia Tai, Chung-Sheng Shi, Shing-Hwa Liu, Pey-Jium Chang, Chung-Cheng Wang, Kuo-How Huang

**Affiliations:** 1 Graduate Institute of Clinical Medical Sciences, College of Medicine, Chang Gung University, Taoyuan, Taiwan; 2 Department of Neurosurgery, New Taipei Municipal TuCheng Hospital (Built and Operated by Chang Gung Medical Foundation), New Taipei City, Taiwan; 3 Department of Anesthesiology, Far-Eastern Memorial Hospital, New Taipei City, Taiwan; 4 Graduate School of Biotechnology and Bioengineering, College of Medicine and Nursing, Yuan Ze University, Taoyuan, Taiwan; 5 Department of Urology, College of Medicine, National Taiwan University Hospital, Taipei, Taiwan; 6 Graduate Institute of Toxicology, College of Medicine, National Taiwan University, Taipei, Taiwan; 7 Department of Urology, National Taiwan University Hospital Hsin-Chu Branch, Hsin-Chu, Taiwan; 8 Department of Pathology, College of Medicine, National Taiwan University Hospital, National Taiwan University, Taipei, Taiwan; 9 Taipei European School, Taipei, Taiwan; 10 Department of Nephrology, Chang Gung Memorial Hospital, Chiayi, Taiwan; 11 Department of Urology, En Chu Kong Hospital, College of Medicine, National Taiwan University, New Taipei City, Taiwan

**Keywords:** apoptosis, cisplatin, neuroblastoma, PCNA, proteasome-associated deubiquitinating enzymes, VLX1570

## Abstract

Despite intensive multimodal therapy, high-risk neuroblastoma remains associated with poor clinical outcomes because of treatment resistance, recurrence, and progressive disease, underscoring the need for new therapeutic strategies. We investigated whether proteasome-associated deubiquitinating enzymes are therapeutically relevant in neuroblastoma by assessing ubiquitin C-terminal hydrolase L5 (UCHL5) and ubiquitin-specific protease 14 (USP14) expression in neuroblastoma tissues and evaluating the antitumor activity of VLX1570 in preclinical models. Immunohistochemical (IHC) analysis showed stronger UCHL5 and USP14 immunoreactivity in neuroblastoma tissues than in normal peripheral nerve tissue. In human neuroblastoma cell lines IMR-32, SK-N-SH, and SH-SY5Y, VLX1570 reduced cell viability in a dose- and time-dependent manner, induced apoptosis, and triggered G2/M arrest. These effects were accompanied by induction of CCAAT/enhancer-binding protein homologous protein (CHOP) and suppression of proliferating cell nuclear antigen (PCNA)-associated proliferative signaling, as reflected by reduced expression of PCNA, phospho-histone H3, and Bcl-2 together with increased p21 and p53 expression. PCNA knockdown experiments further supported inhibition of PCNA-associated proliferative signaling as a functionally relevant component of VLX1570-induced cytotoxicity. In addition, VLX1570 enhanced cisplatin-induced apoptosis *in vitro* and potentiated cisplatin antitumor activity in neuroblastoma xenograft models. Together, these findings support proteasome-associated deubiquitinating enzyme inhibition as a pharmacologic strategy in neuroblastoma and provide a rationale for further evaluation of VLX1570, particularly in combination with cisplatin.

## Introduction

Neuroblastoma is the most common extracranial solid malignancy of childhood and accounts for a disproportionate fraction of pediatric cancer-related deaths (Maris et al., 2007; Matthay et al., 2016). Despite advances in risk-adapted multimodal therapy, the prognosis of high-risk neuroblastoma remains poor because of treatment resistance, disease recurrence, and progressive metastatic burden (Cheung and Dyer, 2013; Pinto et al., 2015; Krystal and Foster, 2023). Current treatment for high-risk neuroblastoma includes induction chemotherapy, surgery, radiotherapy, consolidation with high-dose chemotherapy and autologous stem cell rescue, differentiation therapy, and anti-GD2-based immunotherapy (Pinto et al., 2015; Smith and Foster, 2018). Although anti-GD2 immunotherapy has improved outcomes and is now an established component of modern treatment (Yu et al., 2010; Krystal and Foster, 2023), chemotherapy remains indispensable as the backbone of frontline treatment and as a key platform for salvage or chemoimmunotherapy regimens in relapsed or refractory disease (Mody et al., 2017; Mody et al., 2020; Wieczorek et al., 2023). These clinical limitations highlight the need to identify mechanism-based therapeutic vulnerabilities that regulate both tumor cell survival and treatment responsiveness in neuroblastoma (Ruggiero et al., 2013; Bagatell et al., 2023; Wahba et al., 2023).

The ubiquitin-proteasome system is a central regulator of protein turnover and cellular proteostasis (Kim et al., 2023). In malignant cells, this system contributes to the control of proliferation, stress adaptation, DNA damage responses, and apoptosis (D'Arcy et al., 2015; Kim et al., 2023). Because cancer cells are often highly dependent on protein quality-control pathways, multiple components of the ubiquitin-proteasome system have been investigated as therapeutic targets across solid and hematologic malignancies (Gutierrez-Diaz et al., 2020; Kim et al., 2023). Among these components, the proteasome-associated deubiquitinating enzymes USP14 and UCHL5, located in the 19S regulatory particle, remove ubiquitin chains from proteasome-bound substrates and thereby modulate proteasomal processing (D'Arcy et al., 2015; Lange et al., 2022). Aberrant activity of these enzymes may facilitate tumor cell survival by preserving proteins that support proliferation and resistance to cell death (Lange et al., 2022). Accordingly, small-molecule inhibition of proteasome-associated deubiquitinating enzymes has attracted interest as an alternative strategy for disrupting proteostasis in cancer cells (D'Arcy et al., 2011; Wang et al., 2015; Lee et al., 2019).

VLX1570 is a proteasome-associated deubiquitinating enzyme inhibitor that has shown cytotoxic activity in several preclinical cancer models (Paulus et al., 2016; Wang et al., 2016). Recent work has further highlighted the ubiquitin-proteasome system as a promising therapeutic axis in neuroblastoma, supporting continued investigation of proteasome- and deubiquitination-targeted strategies in this disease (He et al., 2024). However, its biologic and therapeutic relevance in neuroblastoma has not been adequately defined, although inhibition of USP14 has shown antitumor activity in neuroblastoma models (Yu et al., 2019). In the present study, we first examined the expression of UCHL5 and USP14 in neuroblastoma tissues. We then evaluated the effects of VLX1570 on cell viability, apoptosis, cell-cycle distribution, and cisplatin responsiveness in IMR-32, SK-N-SH, and SH-SY5Y cells, and further assessed its antitumor activity in xenograft models. Given the continued importance of chemotherapy in both frontline and relapsed neuroblastoma treatment, particular attention was directed toward whether VLX1570 could enhance cisplatin antitumor activity while exerting direct cytotoxic effects of its own. Our aim was to determine whether inhibition of proteasome-associated deubiquitinating enzymes represents a plausible therapeutic strategy in neuroblastoma.

## Methods

### Cell lines and culture conditions

Human neuroblastoma cell lines IMR-32, SK-N-SH, and SH-SY5Y were obtained from the American Type Culture Collection (ATCC; Manassas, VA, USA). IMR-32 cells were maintained in Eagle’s Minimum Essential Medium (MEM) supplemented with 10% fetal bovine serum (FBS). SK-N-SH cells were maintained in MEM supplemented with 10% FBS. SH-SY5Y cells were maintained in a 1:1 mixture of MEM and F12 medium supplemented with 10% FBS. All cells were cultured at 37 °C in a humidified atmosphere containing 5% CO2. All culture media, fetal bovine serum (FBS), and supplements were purchased from Corning (Corning Life Sciences, Tewksbury, MA, USA). IMR-32 was used as a MYCN-amplified neuroblastic model, whereas SK-N-SH and SH-SY5Y represented related but distinct non-MYCN-amplified neuroblastoma cell contexts.

### Reagents, compounds, and antibodies

VLX1570 was purchased from MedChemExpress (MCE; Monmouth Junction, NJ, USA). Cisplatin was used as the clinical formulation Kemoplat Injection (Fresenius Kabi Taiwan Ltd., Taipei, Taiwan; manufactured by Fresenius Kabi Oncology Ltd., Solan, India) and was prepared according to the manufacturer’s recommendations. For cell-based experiments, the treatment vehicle consisted of 45% normal saline, 40% polyethylene glycol 300 (PEG300), 10% dimethyl sulfoxide (DMSO), and 5% Tween-80. Vehicle control groups received the corresponding vehicle formulation, and the final vehicle concentration was kept constant across treatment groups. Primary antibodies used in this study included UCHL5 (#11527-1-AP; Proteintech Group, Rosemont, IL, USA); USP14 (#MA5-32821; Thermo Fisher Scientific, Waltham, MA, USA); CHOP (#2895), caspase-3 (#14220), caspase-7 (#12827), poly(ADP-ribose) polymerase (PARP; #9542), cleaved caspase-3 (#9664), cleaved caspase-7 (#8438), cleaved PARP (#9541), Bcl-2 (#15071), PCNA (#13110), phospho-histone H3 (#53348), and phospho-histone H2A.X (Ser139) (#9718) (all from Cell Signaling Technology, Danvers, MA, USA); β-actin (#sc-69879), p21 (#sc-397), and p53 (#sc-126) (all from Santa Cruz Biotechnology, Dallas, TX, USA); and glyceraldehyde-3-phosphate dehydrogenase (GAPDH; #GTX100118; GeneTex, Irvine, CA, USA).

### Tissue array analysis

A human neuroblastoma tissue array (catalog number: NB642c; TissueArray.Com LLC, Derwood, MD, USA) was used for IHC analysis. Tissue sections were stained with antibodies against UCHL5 and USP14. Semi-quantitative IHC scoring was performed by a board-certified pathologist (Dr. Wei-Chou Lin, a co-author of this study). Immunoreactivity was scored by multiplying the percentage of positive cells (P) by staining intensity (I) to generate a Q score (Q = P × I). The percentage of positive cells was scored as 0, 0%; 1, 1%–10%; 2, 11%–50%; 3, 51%–80%; and 4, >80%. Staining intensity was scored as 0, absent; 1, weak; 2, moderate; and 3, strong.

### Cell viability assay

For viability assays, 3–5 × 10^3^ cells were seeded per well in 96-well plates and treated with the indicated treatments for 24 or 48 h. Cell viability was assessed using the MTT assay, in which 0.5 mg/mL MTT solution (Sigma-Aldrich, St. Louis, MO, USA) was added to each well and incubated for 4 h at 37 °C. Formazan crystals were subsequently dissolved in dimethyl sulfoxide (DMSO), and absorbance was measured at 570 nm using a microplate reader. Relative viability was normalized to the untreated control group.

### Apoptosis analysis

Apoptosis was quantified using the Muse Annexin V and Dead Cell Assay Kit according to the manufacturer’s instructions. Stained cells were analyzed using a Muse® Cell Analyzer flow cytometer (Merck Millipore, Burlington, MA, USA) with Muse Analysis software. Total apoptosis was defined as the sum of early and late apoptotic fractions (Chow et al., 2020).

### Cell-cycle analysis

Cell-cycle distribution was examined by propidium iodide (PI) staining and flow cytometry. Neuroblastoma cells were treated with VLX1570 or DMSO for 48 h, then fixed in 70% ethanol at −20 °C and stained with PI/RNase A solution (Merck Millipore, Burlington, MA, USA). DNA content was analyzed using a Muse® Cell Analyzer flow cytometer (Merck Millipore, Burlington, MA, USA), and the fractions of cells in G0/G1, S, and G2/M phases were quantified according to the manufacturer’s guidelines.

### Western blot analysis

Cells were lysed in RIPA buffer containing protease and phosphatase inhibitors (Sigma-Aldrich, St. Louis, MO, USA). Equal amounts of protein (20–40 μg) were resolved by SDS-PAGE and transferred to polyvinylidene fluoride (PVDF) membranes (Merck Millipore, Burlington, MA, USA). Membranes were blocked and incubated overnight at 4 °C with primary antibodies, followed by HRP-conjugated secondary antibodies for 1 h at room temperature. Protein bands were detected by chemiluminescence (Kuo et al., 2015). Full, uncropped Western blot images are provided in the Supplementary Figures.

### PCNA knockdown

For PCNA knockdown, neuroblastoma cells were transfected with 10 nM SMARTpool siRNA targeting PCNA (Thermo Scientific Dharmacon, Lafayette, CO, USA) or 10 nM nontargeting scrambled siRNA as a control by using DharmaFECT 1 transfection reagent (Thermo Scientific Dharmacon) in accordance with the manufacturer’s instructions. Subsequently, the transfected cells were cultured with or without VLX1570 in complete medium for 24 h, followed by cell viability analysis.

### Xenograft experiments

Male nude mice (CAnN.Cg-Foxn1nu/CrlNarl; 8 weeks old) were obtained from the Taiwan National Center for Biomodels (Taipei, Taiwan). Neuroblastoma xenografts were established by subcutaneous implantation of 5 × 10^6^ IMR-32 or SK-N-SH cells in Matrigel® Basement Membrane Matrix (Corning, #354234). After tumor establishment, mice were randomized to vehicle, VLX1570, cisplatin, or combination-treatment groups (n ≥ 5 per arm). VLX1570 was administered intraperitoneally (i.p.) at 3.0 mg/kg twice per week, whereas cisplatin was administered intraperitoneally (i.p.) at 5 mg/kg once per week. The treatment vehicle consisted of 45% normal saline, 40% polyethylene glycol 300 (PEG300), 10% DMSO, and 5% Tween-80. Tumor volumes were measured twice weekly using calipers and calculated as (length × width^2^)/2. Mice were monitored for body weight and signs of toxicity throughout the study. After 5 weeks of treatment, mice were euthanized and tumors were excised, photographed, and weighed. All animal procedures were approved by the Institutional Animal Care and Use Committee (IACUC approval no. 20240129). Humane endpoints followed institutional policy, including maximal tumor diameter ≤15 mm or tumor volume ≤1500 mm^3^, absence of ulceration or necrosis, and body-weight loss <15%; no animal exceeded these limits during the study.

### Statistical analysis

Data are presented as mean ± SD. Comparisons between two groups were analyzed using Student’s *t*-test, whereas comparisons among multiple groups were analyzed using one-way ANOVA followed by Tukey’s *post hoc* test. A *p* value of <0.05 was considered statistically significant.

## Results

### UCHL5 and USP14 are overexpressed in neuroblastoma tissues

**FIGURE 1:**
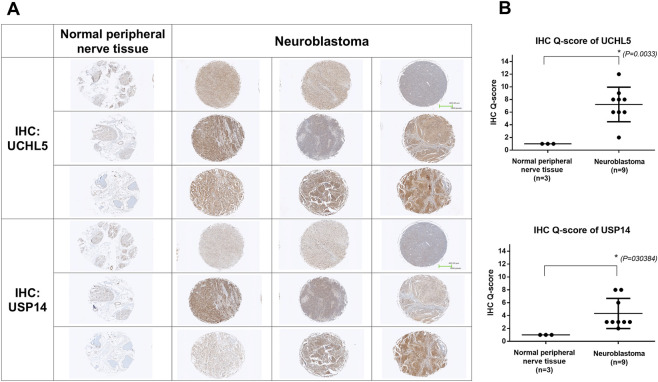
UCHL5 and USP14 are overexpressed in neuroblastoma tissues. **(A)** IHC staining of UCHL5 and USP14 in a human neuroblastoma tissue array. Relative to normal peripheral nerve tissue, neuroblastoma tissues exhibited stronger immunoreactivity for both UCHL5 and USP14. Representative images are shown. **(B)** Semi-quantitative IHC Q-score analysis of UCHL5 and USP14 expression in normal peripheral nerve tissue and neuroblastoma tissue. Q scores were calculated by multiplying the percentage of positive cells by staining intensity. Statistical analysis was performed using Student’s t-test.

We first examined UCHL5 and USP14 protein expression in clinical specimens by immunohistochemistry. Relative to normal peripheral nerve tissue, neuroblastoma tissues exhibited stronger immunoreactivity for both UCHL5 and USP14 (Figure 1A). Semi-quantitative IHC Q-score analysis further confirmed that the Q scores for both UCHL5 and USP14 were significantly higher in neuroblastoma tissues than in normal peripheral nerve tissue (Figure 1B). These findings indicate that UCHL5 and USP14 are upregulated in neuroblastoma tissues and support further evaluation of VLX1570 in neuroblastoma models.

### VLX1570 decreases cell viability and induces apoptosis in neuroblastoma cells

**FIGURE 2:**
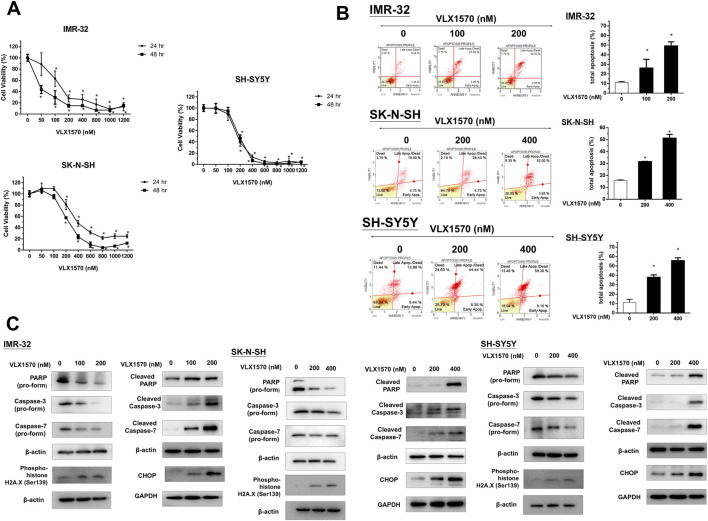
VLX1570 decreases cell viability and induces apoptosis in neuroblastoma cells. **(A)** Cell viability of IMR-32, SK-N-SH, and SH-SY5Y cells after treatment with VLX1570 at the indicated concentrations for 24 or 48 h, as determined by MTT assay. **(B)** Representative flow cytometry plots and quantification of total apoptosis in IMR-32, SK-N-SH, and SH-SY5Y cells after VLX1570 treatment. Total apoptosis was defined as the sum of early and late apoptotic fractions. **(C)** Western blot analysis of CHOP, cleaved PARP, cleaved caspase-3, cleaved caspase-7, pro-form PARP, pro-form caspase-3, pro-form caspase-7, and phospho-histone H2A.X (Ser139) in neuroblastoma cells after VLX1570 treatment. Data are presented as mean ± SD from three independent experiments. *p < 0.05.

We next evaluated the cytotoxic effects of VLX1570 in IMR-32, SK-N-SH, and SH-SY5Y neuroblastoma cells. VLX1570 reduced cell viability in all three cell lines in a dose- and time-dependent manner (Figure 2A). Flow cytometric analysis, including representative apoptosis plots and quantitative analysis, showed a corresponding increase in apoptotic cells following treatment (Figure 2B). Consistent with these findings, Western blot analysis demonstrated increased cleavage of PARP, caspase-3, and caspase-7 after VLX1570 exposure, accompanied by decreased expression of the corresponding pro-forms (Figure 2C). Induction of CHOP was also observed under the same treatment conditions. In addition, phospho-histone H2A.X (Ser139) expression increased following VLX1570 treatment, consistent with enhanced DNA damage signaling (Figure 2C). Together, these findings indicate that VLX1570 induces apoptotic cell death in neuroblastoma cells and is associated with activation of endoplasmic reticulum stress- and DNA damage-related responses (Barbagallo et al., 2019).

### VLX1570 induces G2/M arrest and suppresses PCNA-associated proliferative signaling in neuroblastoma cells

**FIGURE 3:**
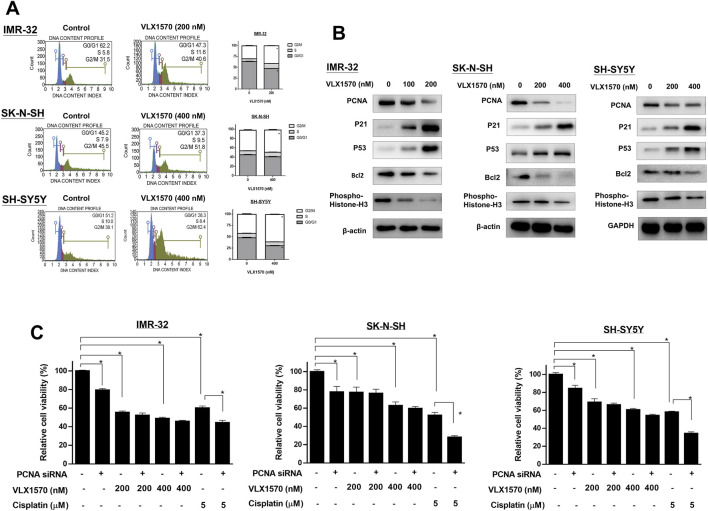
VLX1570 induces G2/M arrest and suppresses PCNA-associated proliferative signaling in neuroblastoma cells. **(A)** Representative cell-cycle profiles and quantification of G0/G1, S, and G2/M populations in IMR-32, SK-N-SH, and SH-SY5Y cells after VLX1570 treatment, showing induction of G2/M arrest. **(B)** Western blot analysis of PCNA, p21, p53, phospho-histone H3, and Bcl-2 in neuroblastoma cells after VLX1570 treatment. **(C)** Relative cell viability of IMR-32, SK-N-SH, and SH-SY5Y cells after transfection with PCNA-targeting siRNA or nontargeting scrambled siRNA control, followed by treatment with VLX1570 (200 or 400 nM) or cisplatin, as indicated. PCNA knockdown alone reduced cell viability in all three cell lines. However, additional PCNA knockdown did not further reduce viability in VLX1570-treated cells, whereas PCNA knockdown further enhanced the viability reduction induced by cisplatin. For groups not receiving PCNA-targeting siRNA, scrambled siRNA was used as the corresponding transfection control. Data are presented as mean ± SD from three independent experiments. *p < 0.05.

To further characterize the biologic effects of VLX1570, we analyzed cell-cycle distribution after treatment. VLX1570 induced G2/M arrest in IMR-32, SK-N-SH, and SH-SY5Y cells, accompanied by an increase in the G2/M fraction and a decrease in the G0/G1 fraction (Figure 3A). Western blot analysis further showed reduced expression of PCNA, phospho-histone H3, and Bcl-2, together with increased p21 and p53 expression (Figure 3B). These findings suggest that, in addition to inducing apoptosis, VLX1570 suppresses PCNA-associated proliferative signaling and disrupts cell-cycle progression in neuroblastoma cells. Taken together, these molecular changes are consistent with a cell-cycle inhibitory response under VLX1570 treatment conditions.

### PCNA suppression contributes to VLX1570-induced cytotoxicity in neuroblastoma cells

To determine whether PCNA suppression contributes functionally to the cytotoxic effect of VLX1570 in neuroblastoma cells, we examined the consequences of PCNA knockdown under VLX1570 treatment conditions. IMR-32, SK-N-SH, and SH-SY5Y cells were transfected with PCNA-targeting siRNA or scrambled siRNA control and then treated with VLX1570 or cisplatin. PCNA knockdown alone reduced cell viability in all three cell lines (Figure 3C). VLX1570 alone at 200 or 400 nM also decreased cell viability. However, under VLX1570 treatment conditions, additional PCNA knockdown did not further reduce viability at either concentration (Figure 3C). This non-additive pattern indicates that PCNA suppression contributes to VLX1570-induced cytotoxicity and supports inhibition of PCNA-associated proliferative signaling as a functionally relevant component of VLX1570 activity. In contrast, PCNA knockdown further enhanced the viability reduction induced by cisplatin (Figure 3C), indicating that cisplatin-mediated cytotoxicity is not fully explained by PCNA suppression alone.

### VLX1570 enhances cisplatin-induced apoptosis in neuroblastoma cells

**FIGURE 4:**
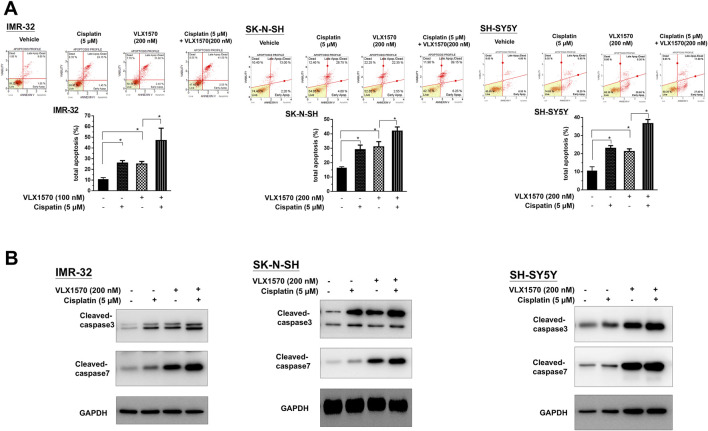
VLX1570 enhances cisplatin-induced apoptosis in neuroblastoma cells. **(A)** Representative flow cytometry plots and quantification of total apoptosis in IMR-32, SK-N-SH, and SH-SY5Y cells treated with VLX1570, cisplatin, or the combination, as indicated. Combined treatment increased the apoptotic fraction relative to either single agent alone in all three cell lines. **(B)** Western blot analysis showing greater cleavage of caspase-3 and caspase-7 after combination treatment than after either monotherapy. Data are presented as mean ± SD from three independent experiments. *p < 0.05.

Given that VLX1570 exhibited direct antitumor activity in neuroblastoma cells, we next examined whether it could enhance the effect of cisplatin, a clinically relevant chemotherapeutic agent. In IMR-32, SK-N-SH, and SH-SY5Y cells, combined treatment with VLX1570 and cisplatin increased the apoptotic fraction relative to either single agent alone (Figure 4A). Western blot analysis further demonstrated greater cleavage of caspase-3 and caspase-7 in the combination group than in the monotherapy groups (Figure 4B). These findings indicate that VLX1570 enhances cisplatin-induced apoptosis in neuroblastoma cells.

### VLX1570 combined with cisplatin suppresses neuroblastoma xenograft growth more effectively than monotherapy

**FIGURE 5:**
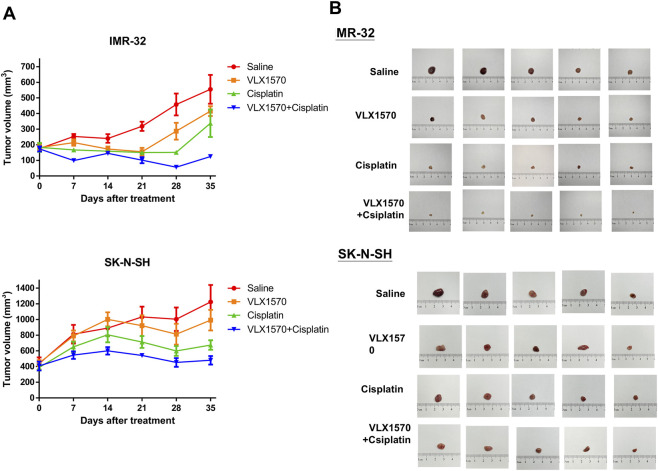
VLX1570 potentiates cisplatin antitumor activity in neuroblastoma xenograft models. **(A)** Tumor growth curves for IMR-32 xenografts treated with vehicle, VLX1570, cisplatin, or VLX1570 plus cisplatin. **(B)** Tumor growth curves for SK-N-SH xenografts under the same treatment conditions. In both xenograft models, the combination regimen produced greater tumor growth inhibition than either monotherapy. Tumor growth data are presented as mean ± SEM.

To determine whether the enhanced activity observed *in vitro* was recapitulated *in vivo*, we evaluated VLX1570 in neuroblastoma xenograft models. In both models, VLX1570 or cisplatin monotherapy attenuated tumor growth to varying degrees, whereas the combination regimen produced the greatest suppression of tumor progression over the course of treatment (Figures 5A,B). These findings indicate that the enhanced antitumor effect observed with VLX1570 and cisplatin *in vitro* was reproduced *in vivo*.

**FIGURE 6:**
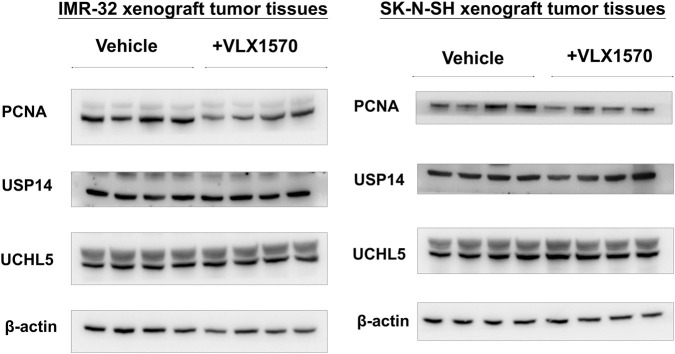
VLX1570 reduces PCNA expression in neuroblastoma xenograft tumor tissues without altering USP14 or UCHL5 expression. Western blot analysis of PCNA, USP14, and UCHL5 expression in tumor tissues collected from vehicle- and VLX1570-treated IMR-32 and SK-N-SH neuroblastoma xenografts. In both xenograft models, tumor tissues from the VLX1570-treated group showed reduced PCNA expression compared with the vehicle group, whereas USP14 and UCHL5 expression levels were not obviously altered. β-actin was used as the loading control.

### VLX1570 reduces PCNA expression in neuroblastoma xenograft tumor tissues without altering USP14 or UCHL5 expression

To further determine whether the molecular changes observed *in vitro* were also present *in vivo*, we performed Western blot analysis on tumor tissues collected from vehicle- and VLX1570-treated neuroblastoma xenografts. In both IMR-32 and SK-N-SH xenograft tumor tissues, VLX1570 treatment was associated with reduced PCNA expression compared with the vehicle group. In contrast, USP14 and UCHL5 expression levels were not obviously altered by VLX1570 treatment. These findings indicate that VLX1570-associated PCNA downregulation *in vivo* was not accompanied by reduced expression of these proteasome-associated deubiquitinating enzymes.

## Discussion

The present study provides preclinical support for proteasome-associated deubiquitinating enzymes as therapeutic targets in neuroblastoma. UCHL5 and USP14 were more strongly expressed in neuroblastoma tissues than in normal peripheral nerve tissue, providing a biologic rationale for pharmacologic evaluation of VLX1570. In parallel, VLX1570 showed direct antitumor activity in neuroblastoma models, reducing cell viability, inducing apoptosis, and potentiating cisplatin antitumor activity.

The direct antitumor activity of VLX1570 was supported by both flow cytometric and Western blot data. VLX1570 reduced cell viability in IMR-32, SK-N-SH, and SH-SY5Y cells and increased apoptotic cell death, accompanied by cleavage of PARP, caspase-3, and caspase-7 together with decreased expression of their corresponding pro-forms. Induction of CHOP under the same treatment conditions suggests that perturbation of proteostasis and activation of an endoplasmic reticulum stress-associated response contribute to this effect (Barbagallo et al., 2019). In addition, increased phospho-histone H2A.X (Ser139) expression following VLX1570 treatment is consistent with enhanced DNA damage signaling. This interpretation is consistent with previous studies showing that inhibition of proteasome-associated deubiquitinating enzymes can trigger proteotoxic stress and apoptotic signaling in malignant cells (Wang et al., 2016; Kurozumi et al., 2021; Wang et al., 2022).

In addition to apoptosis, VLX1570 induced G2/M arrest and reduced PCNA, phospho-histone H3, and Bcl-2 expression together with increased p21 and p53 expression. These molecular changes indicate that VLX1570 affects both survival and proliferative programs in neuroblastoma cells. In particular, the reduction in PCNA expression, together with the results of the PCNA knockdown experiments, supports the interpretation that suppression of PCNA-associated proliferative signaling is a functionally relevant component of VLX1570 activity. The non-additive effect observed after combined VLX1570 treatment and PCNA knockdown indicates that PCNA suppression contributes to VLX1570-induced cytotoxicity. At the same time, the finding that VLX1570 plus PCNA knockdown remained more cytotoxic than PCNA knockdown alone suggests that VLX1570 does not act exclusively through the PCNA axis. Additional mechanisms, potentially including ER stress-associated apoptosis as suggested by induction of CHOP, are therefore also likely to contribute to the overall antitumor effect.

An additional strength of the current dataset is that these effects were observed across IMR-32, SK-N-SH, and SH-SY5Y cells rather than in a single neuroblastoma model. This is relevant because neuroblastoma is biologically heterogeneous, and therapeutic effects restricted to one cellular context are often difficult to generalize (Cheung and Dyer, 2013; Bagatell et al., 2023; Wieczorek et al., 2023). The preservation of VLX1570 activity across these three models supports the view that the observed antitumor effects are not limited to a single neuroblastoma cell background.

The combination data with cisplatin are particularly important from a translational perspective. Chemotherapy remains central to the management of both newly diagnosed high-risk disease and relapsed or refractory neuroblastoma (Mody et al., 2017; Mody et al., 2020; Wieczorek et al., 2023). Agents that can potentiate the activity of clinically relevant chemotherapeutic agents therefore remain of considerable interest. This concept is also supported by prior neuroblastoma work showing that proteasome inhibition with carfilzomib enhances cisplatin-induced apoptosis and maintains additive activity even in cisplatin-resistant cells (Lee et al., 2019). In the present study, VLX1570 enhanced cisplatin-induced apoptosis *in vitro* and potentiated cisplatin antitumor activity in neuroblastoma xenograft models. PCNA knockdown also further sensitized neuroblastoma cells to cisplatin, supporting the interpretation that cisplatin does not exert its cytotoxic effect primarily through PCNA suppression and instead acts through at least partially distinct downstream mechanisms. Together, these findings support the view that VLX1570 and cisplatin affect overlapping but non-identical survival programs in neuroblastoma cells and provide a rationale for evaluating this combination further.

Although the current data do not establish PCNA as a direct molecular substrate of USP14 or UCHL5, they do indicate that PCNA downregulation is functionally linked to VLX1570-induced cytotoxicity. Because PCNA regulation has been more clearly connected to other deubiquitinating enzymes, particularly USP1 (Huang et al., 2006; Simoneau et al., 2023), whether PCNA is regulated indirectly downstream of proteasome-associated deubiquitinating enzyme inhibition or is more closely linked to the UCHL5/USP14 axis remains to be determined. Thus, direct regulation of PCNA by USP14 or UCHL5, as well as direct disruption of deubiquitination-dependent PCNA control by VLX1570, will require additional mechanistic investigation in future studies.

Several limitations should be acknowledged. First, the current dataset does not identify the critical downstream substrates or signaling nodes responsible for the observed phenotype. Second, although UCHL5 and USP14 were both expressed in neuroblastoma tissues, the relative contribution of each enzyme to VLX1570 sensitivity remains unresolved. Third, broader validation in additional neuroblastoma models would further strengthen the generalizability of the conclusions. Finally, although the current findings support a functional link between PCNA suppression and VLX1570-induced cytotoxicity, additional mechanistic studies will be required to define the upstream events responsible for this effect more precisely. In addition, formal histopathologic toxicity evaluation of major organs was not available in the current animal study. Although VLX1570 had previously undergone preclinical toxicology evaluation prior to clinical testing (Rowinsky et al., 2020), the present *in vivo* study did not include organ-level histopathologic assessment, which should be addressed in future translational investigations.

In summary, VLX1570 suppresses neuroblastoma growth, at least in part, through inhibition of PCNA-associated proliferative signaling and induction of apoptosis, and potentiates cisplatin antitumor activity *in vitro* and *in vivo*. These findings support further evaluation of proteasome-associated deubiquitinating enzyme inhibition as a pharmacologic strategy in neuroblastoma, particularly in combination with clinically relevant chemotherapy.

## Data Availability

The original contributions presented in the study are included in the article/Supplementary Material, further inquiries can be directed to the corresponding author/s.

## Supplementary material

Supplementary Figure S1 Uncropped original Western blot images corresponding to Figure 2C for IMR-32 cells.

Supplementary Figure S2 Uncropped original Western blot images corresponding to Figure 2C for SK-N-SH cells.

Supplementary Figure S3 Uncropped original Western blot images corresponding to Figure 2C for SH-SY5Y cells.

Supplementary Figure S4 Uncropped original Western blot images corresponding to Figure 3B for IMR-32 cells.

Supplementary Figure S5 Uncropped original Western blot images corresponding to Figure 3B for SK-N-SH cells.

Supplementary Figure S6 Uncropped original Western blot images corresponding to Figure 3B for SH-SY5Y cells.

Supplementary Figure S7 Uncropped original Western blot images corresponding to Figure 4B for IMR-32 cells.

Supplementary Figure S8 Uncropped original Western blot images corresponding to Figure 4B for SK-N-SH cells.

Supplementary Figure S9 Uncropped original Western blot images corresponding to Figure 4B for SH-SY5Y cells.

Supplementary Figure S10 Uncropped original Western blot images corresponding to Figure 6 for IMR-32 xenograft tumor tissues.

Supplementary Figure S11 Uncropped original Western blot images corresponding to Figure 6 for SK-N-SH xenograft tumor tissues.
