# Supplementary figures and images for: VLX1570 suppresses neuroblastoma growth through inhibition of PCNA-associated proliferative signaling and potentiates cisplatin antitumor activity
Source: Front Pharmacol. 2026 Jul 15;17:1857567. doi: 10.3389/fphar.2026.1857567 (PMC13416542; doi:10.3389/fphar.2026.1857567)

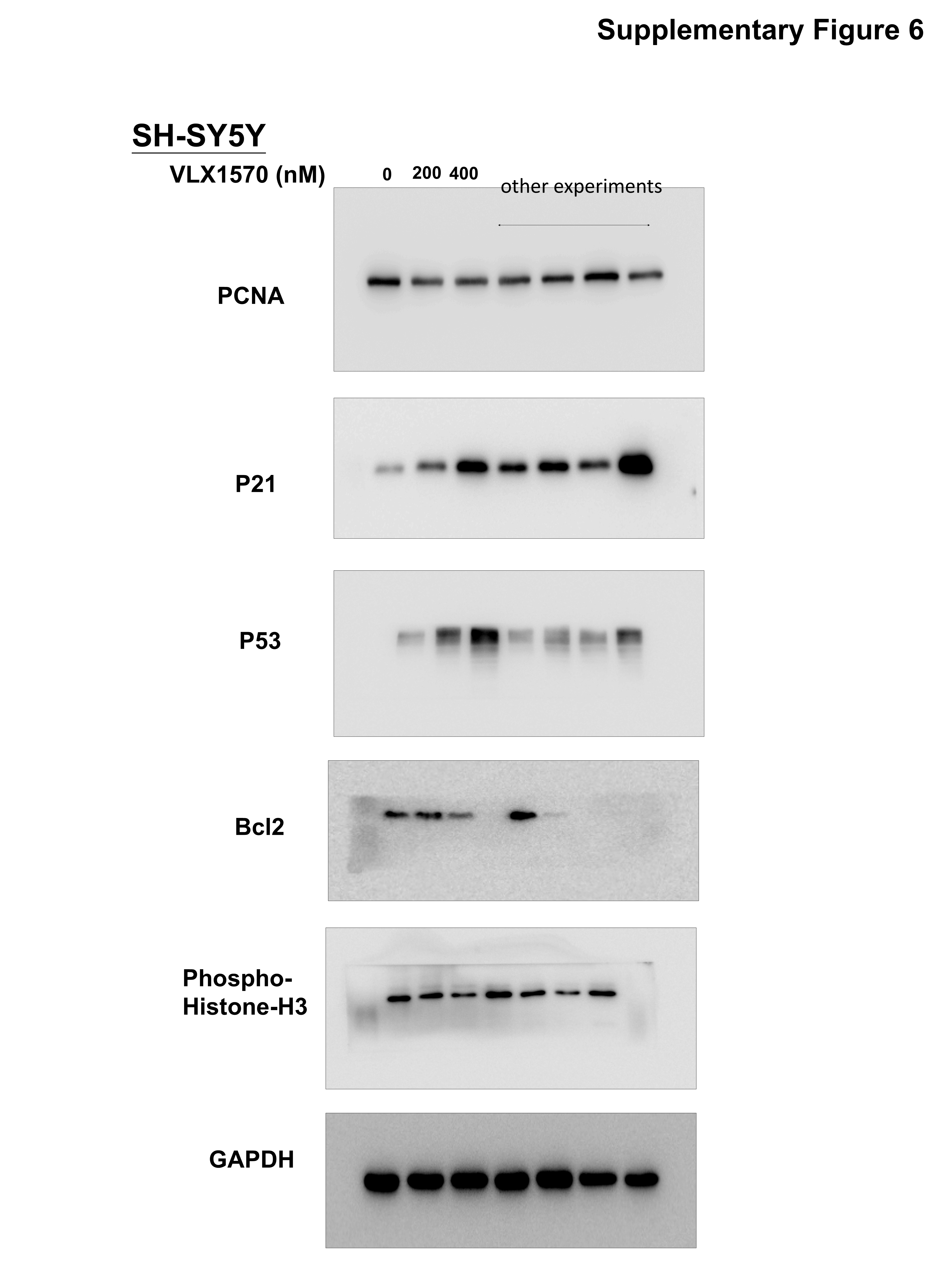

Supplement: Supplementary file 1 [file Image6.tif]

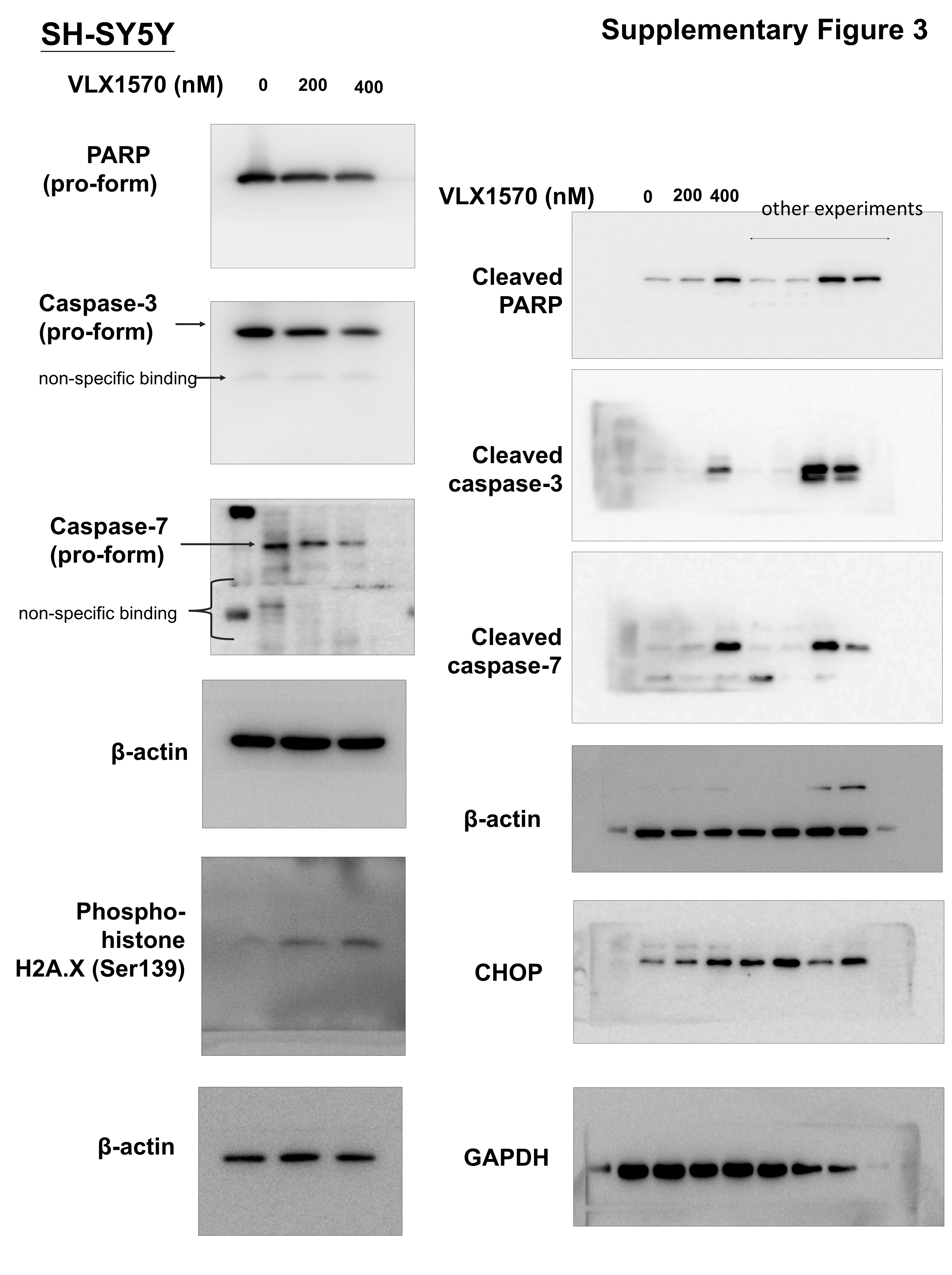

Supplement: Supplementary file 2 [file Image3.tif]

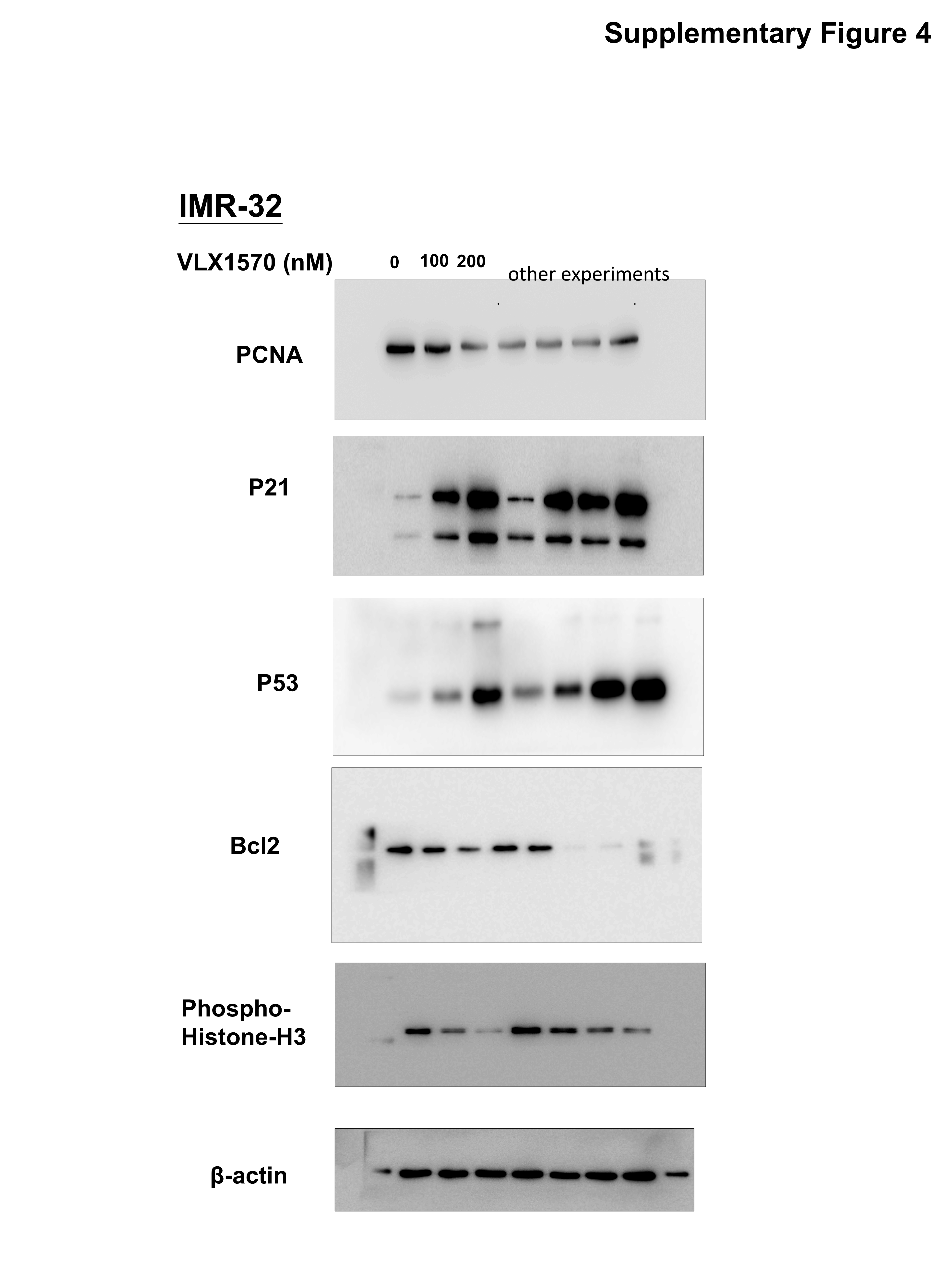

Supplement: Supplementary file 3 [file Image4.tif]

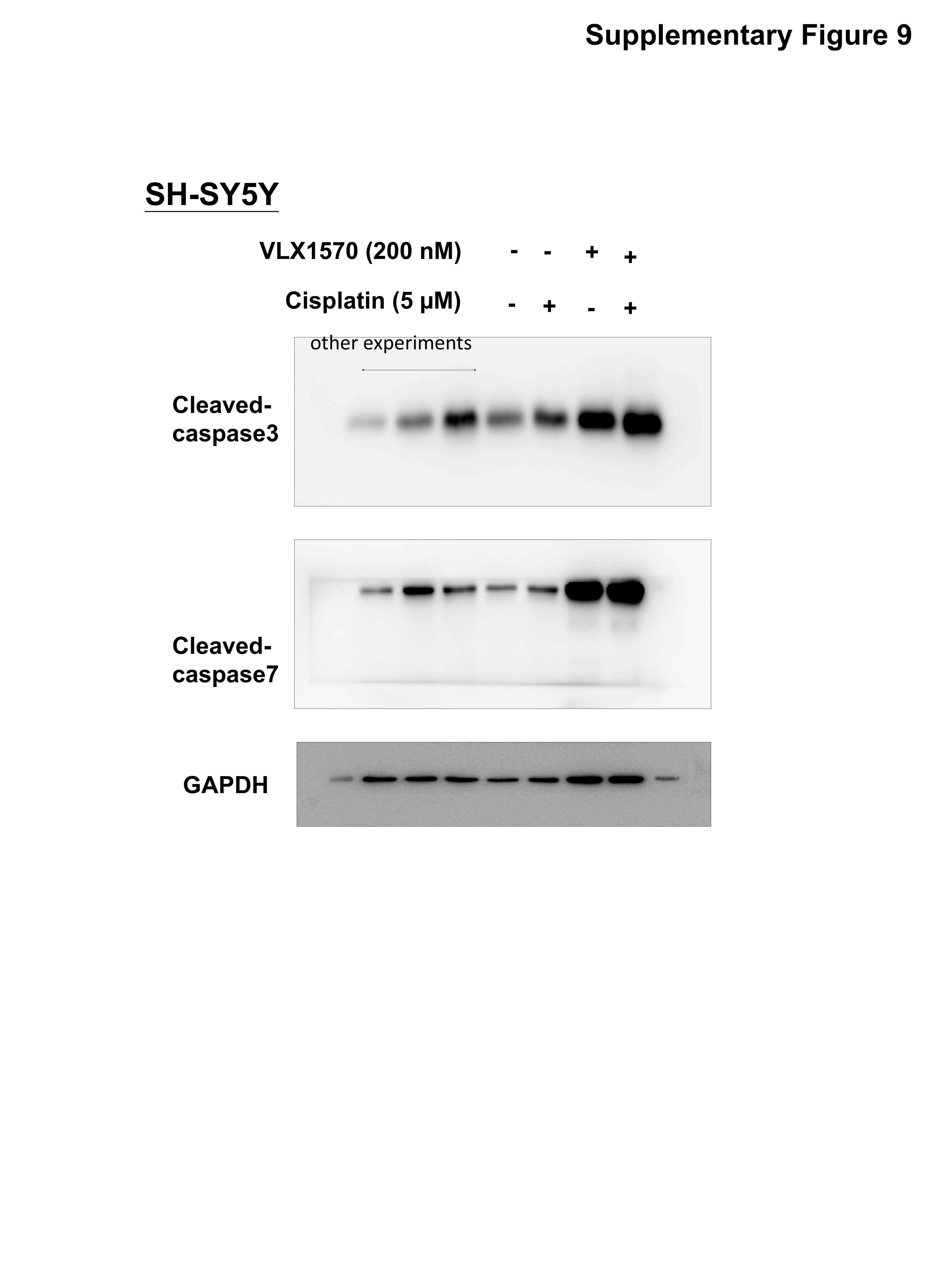

Supplement: Supplementary file 4 [file Image9.tif]

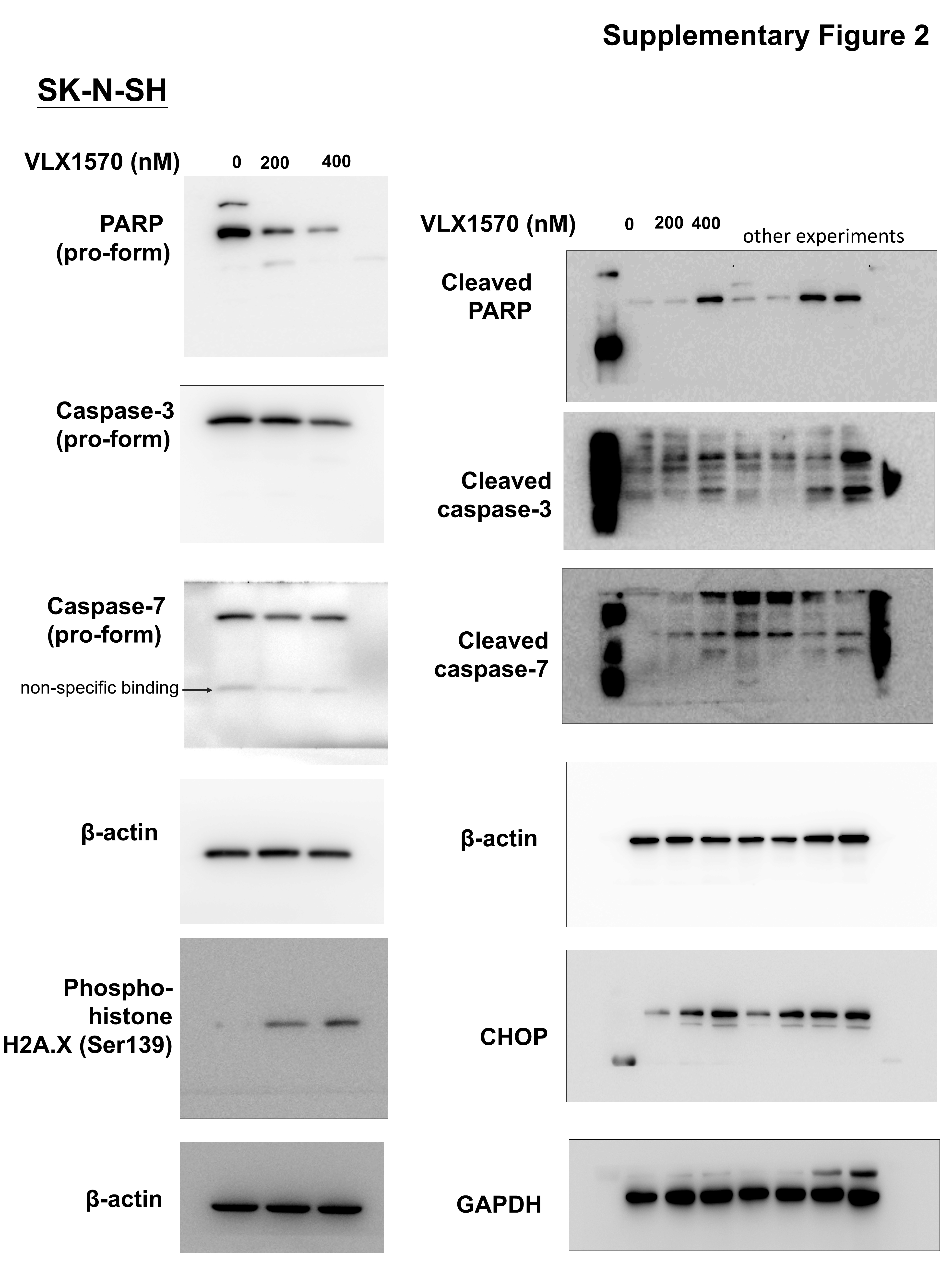

Supplement: Supplementary file 5 [file Image2.tif]

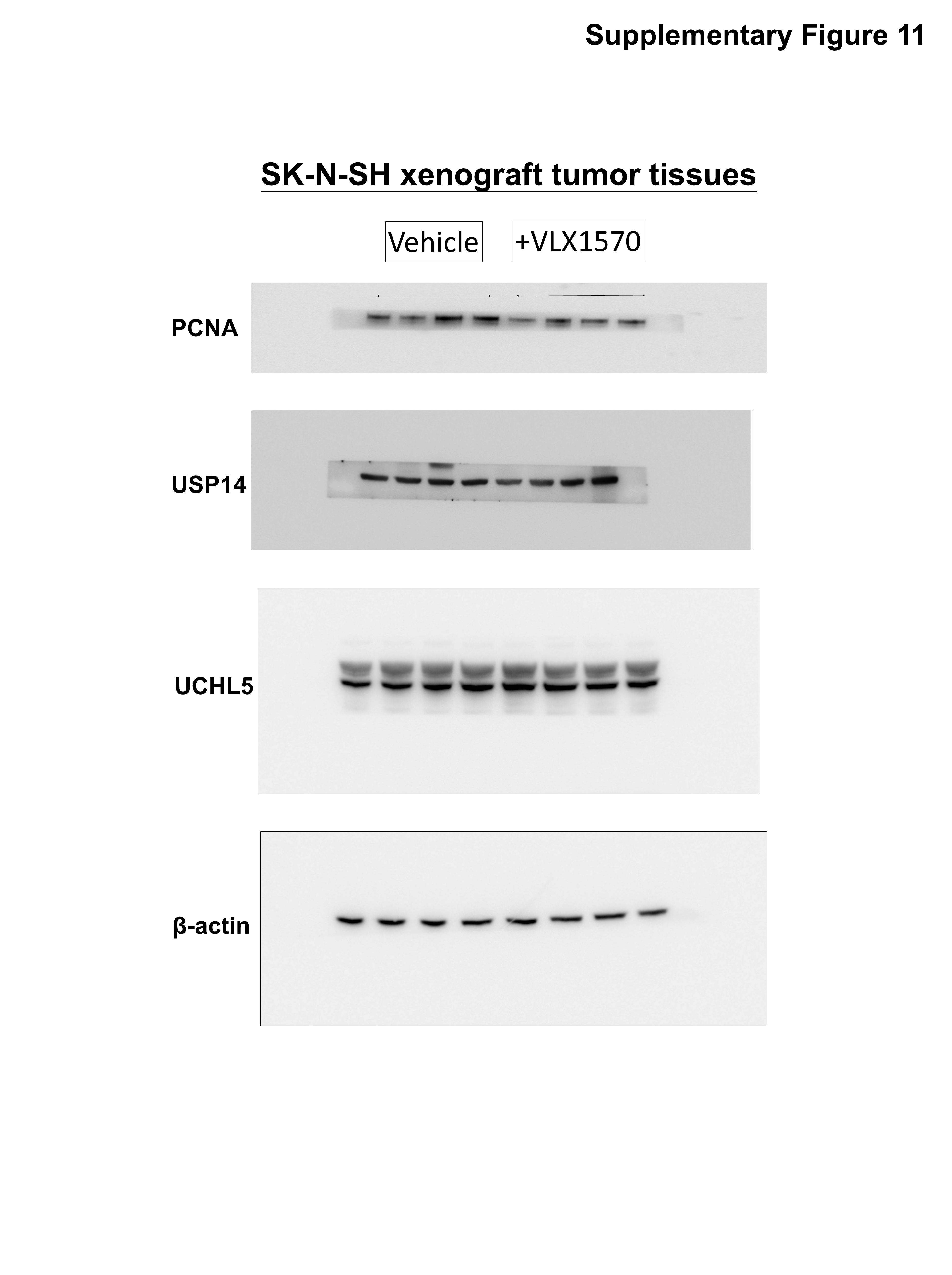

Supplement: Supplementary file 6 [file Image11.tif]

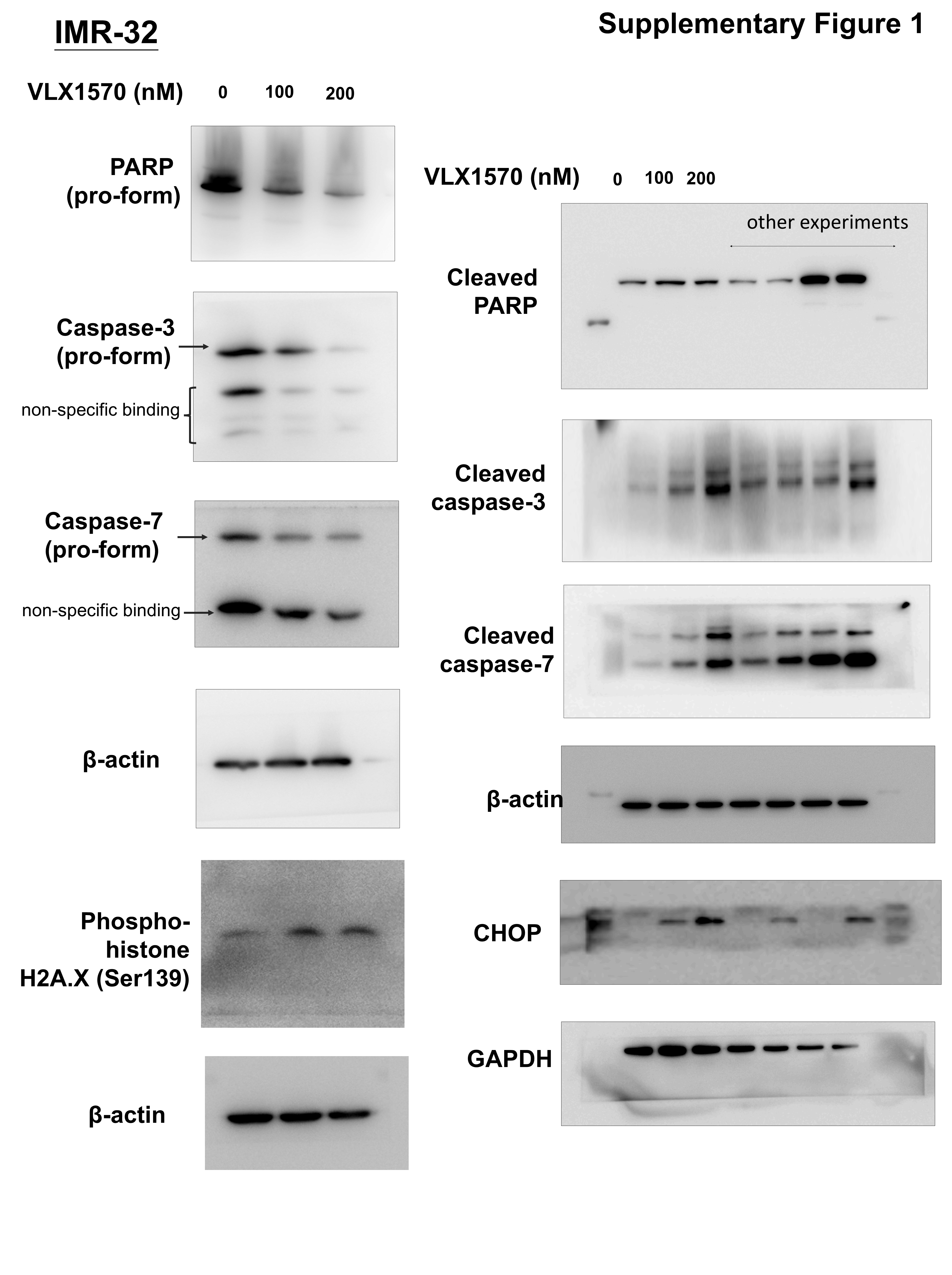

Supplement: Supplementary file 7 [file Image1.tif]

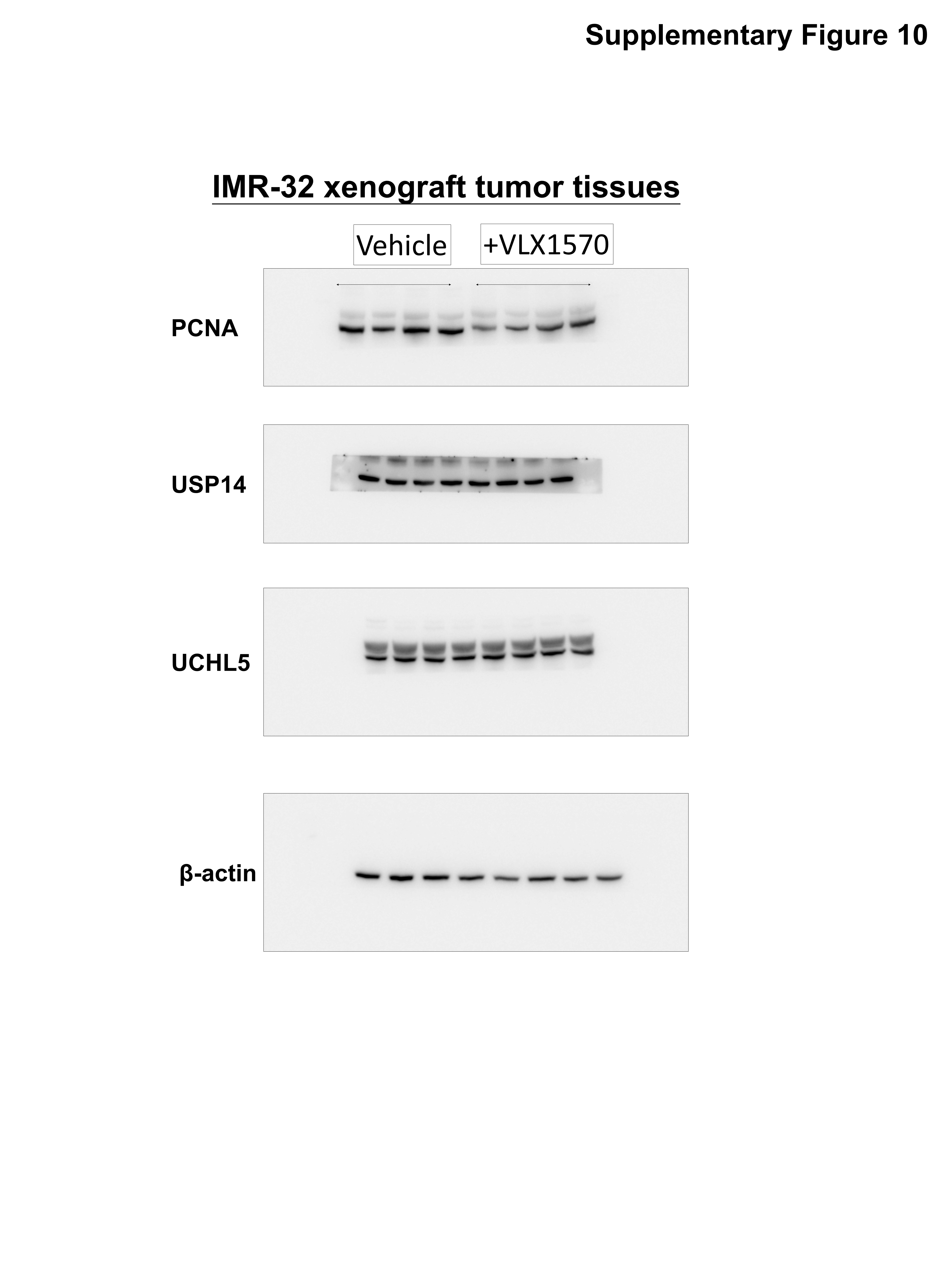

Supplement: Supplementary file 8 [file Image10.tif]

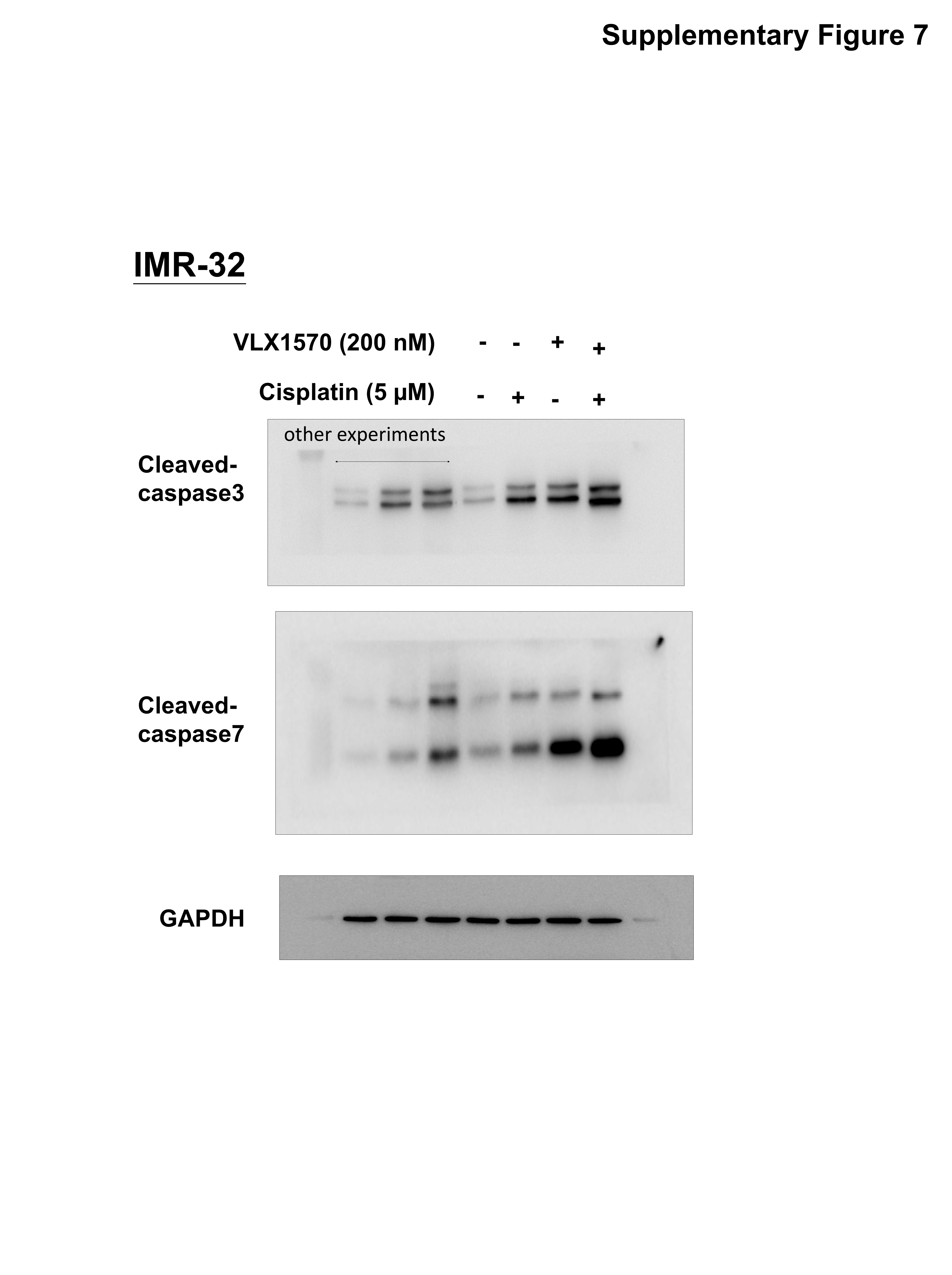

Supplement: Supplementary file 9 [file Image7.tif]

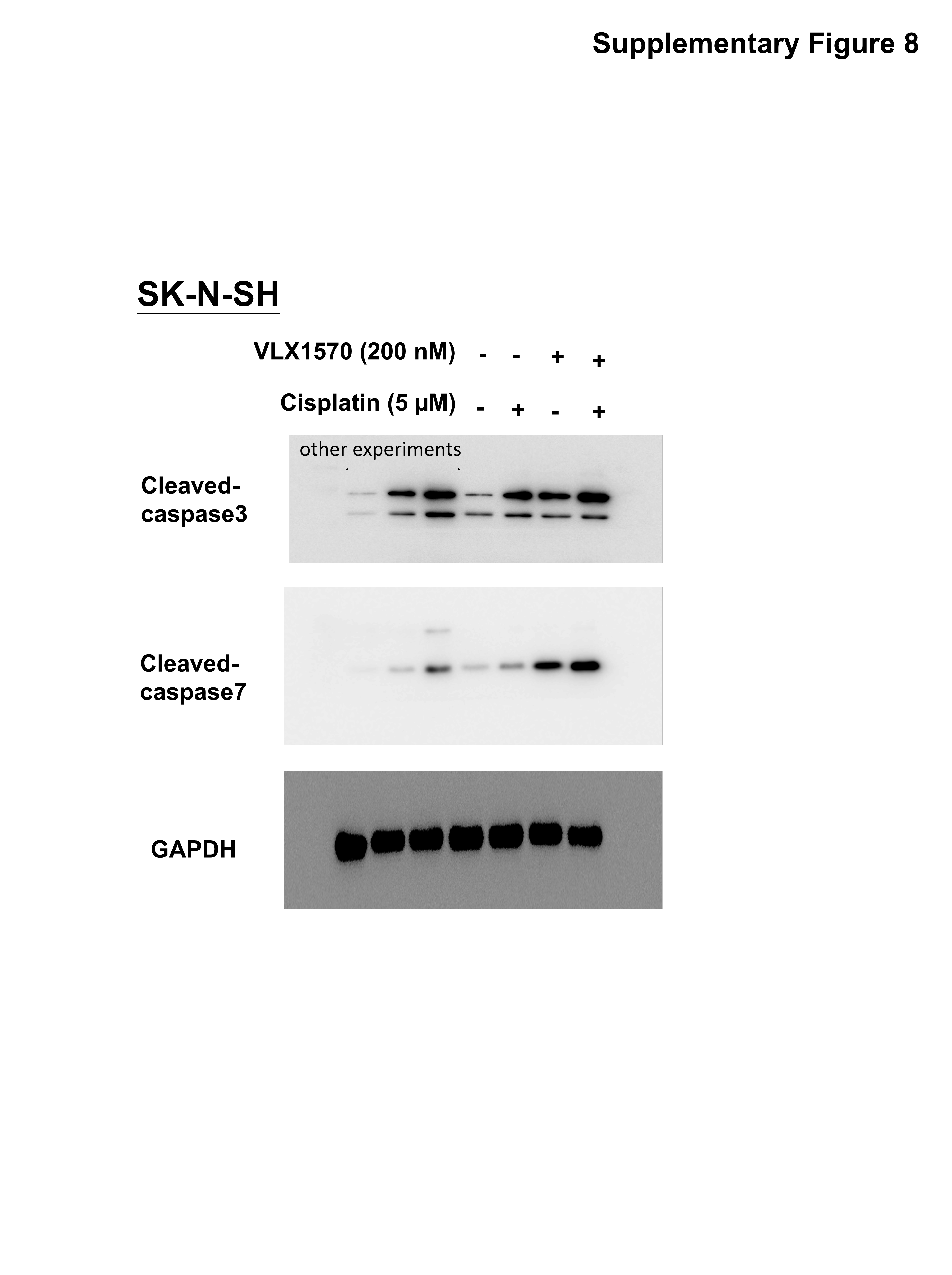

Supplement: Supplementary file 10 [file Image8.tif]

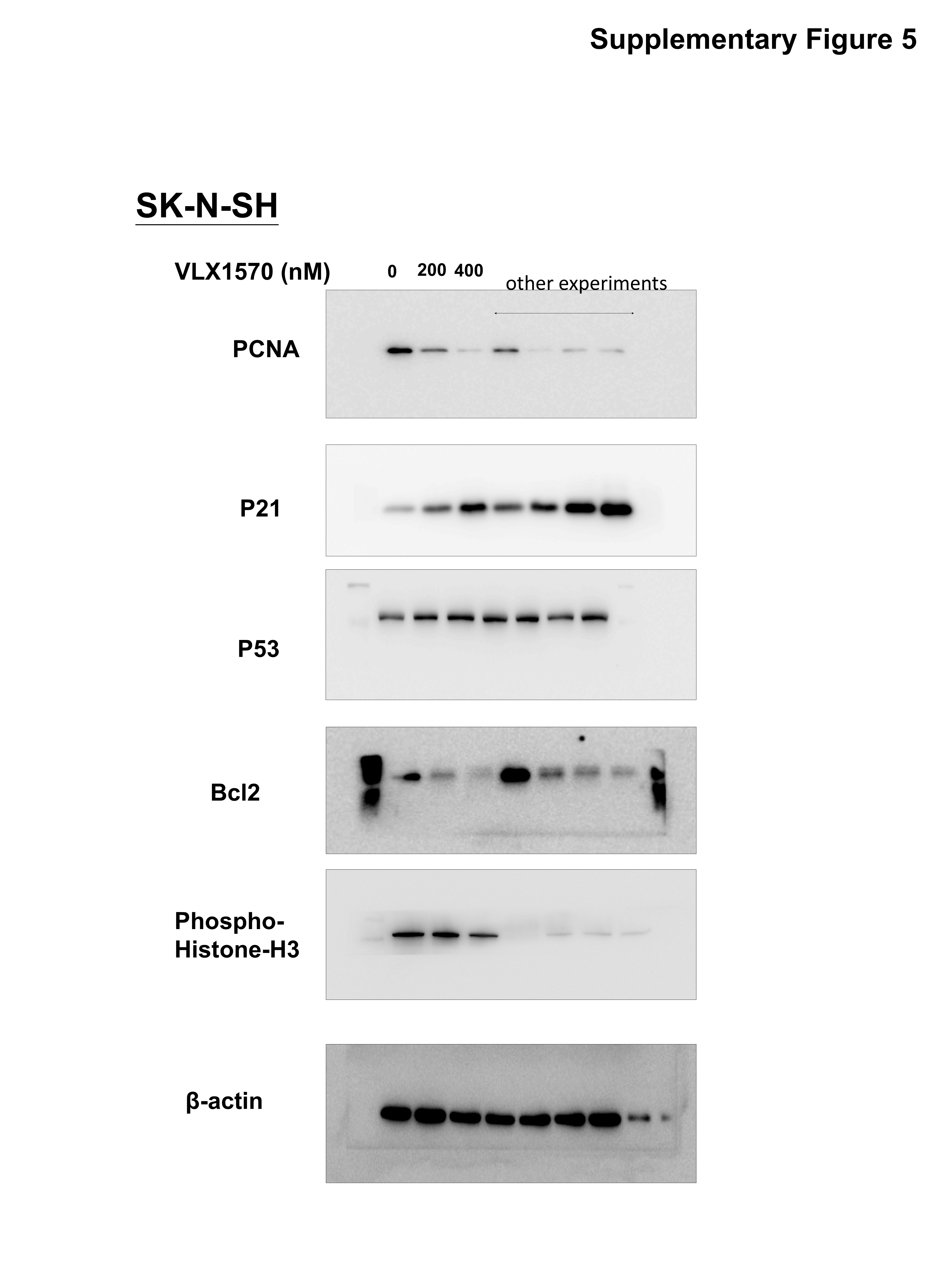

Supplement: Supplementary file 11 [file Image5.tif]
